# Nuclear-enriched abundant transcript 1 as a diagnostic and prognostic biomarker in colorectal cancer

**DOI:** 10.1186/s12943-015-0455-5

**Published:** 2015-11-09

**Authors:** Yuchen Wu, Li Yang, Jiang Zhao, Cong Li, Jia Nie, Fangqi Liu, Changhua Zhuo, Yaxin Zheng, Bin Li, Zhimin Wang, Ye Xu

**Affiliations:** Department of Colorectal Surgery, Fudan University Shanghai Cancer Center; Department of Oncology, Shanghai Medical College, Fudan University, No. 270 Dong-an Road, Shanghai, 20032 People’s Republic of China; Key Laboratory of Molecular Virology & Immunology, Unit of Molecular Immunology, Institut Pasteur of Shanghai, Shanghai Institutes for Biological Sciences, Chinese Academy of Sciences, No. 320 Yue-yang Road, Shanghai, 20031 People’s Republic of China; Department of Surgical Oncology, Fujian Provincial Cancer Hospital, Teaching Hospital of Fujian Medical University, No. 420 Fu-ma Road, Fuzhou, 350014 People’s Republic of China; Eastern Hepatobiliary Hospital, Second Military Medical University, No. 225 Chang-hai Road, Shanghai, 200438 People’s Republic of China; Department of Genetics, Shanghai-MOST Key Laboratory of Health and Disease Genomics, Chinese National Human Genome Center and Shanghai Industrial Technology Institute (SITI), No. 250 Bi-bo Road, Shanghai, 201203 People’s Republic of China

**Keywords:** Biomarker, Prognosis, Diagnosis, Colorectal cancer, Long non-coding RNA

## Abstract

**Background:**

High expression of the long non-coding RNA nuclear-enriched abundant transcript 1 (NEAT1) in whole blood has been reported in colorectal cancer patients; however, its’ clinical significance and origin are unclear. We evaluated the diagnostic and prognostic value, and origin of whole blood NEAT1 in colorectal cancer.

**Methods:**

Expression of NEAT1 variants*,* NEAT1_v1 and NEAT1_v2 were determined using real-time quantitative PCR. The diagnostic value of whole blood NEAT1 expression was evaluated in test (*n* = 60) and validation (*n* = 200) cohorts of colorectal cancer patients and normal controls (NCs). To identify the origin of NEAT1, its expression was analyzed in blood, matched primary tumor tissues, para-tumor tissues, metastatic tissues, and also immune cells from patients or NCs. Function of NEAT1 in colorectal cell lines was also assessed. The correlation of NEAT1 expression with clinical outcomes was assessed in 191 patients.

**Results:**

Whole blood NEAT1 expression was significantly higher in colorectal cancer patients than in NCs. NEAT1_v1 and NEAT1_v2 expression were highly accurate in distinguishing colorectal cancer patients from NCs (area under the curve: 0.787 and 0.871, respectively). Knockdown of NEAT1_v1 in vitro could inhibit cell invasion and proliferation, while knockdown of NEAT1_v2 promoted cell growth. However, whole blood expression was not correlated with matched tissues. An elevated expression was seen in neutrophils from CRC patients. Furthermore, high expression of NEAT1_v1 was correlated with worse overall survival. In contrast, high expression of NEAT1_v2 alone was correlated with better overall survival.

**Conclusion:**

Whole blood NEAT1 expression is a novel diagnostic and prognostic biomarker of overall survival in colorectal cancer. Elevated NEAT1 may derive from neutrophils.

**Electronic supplementary material:**

The online version of this article (doi:10.1186/s12943-015-0455-5) contains supplementary material, which is available to authorized users.

## Background

Current evidence indicates that RNAs play important roles in oncogenesis and cancer pathogenesis. Considering that protein-coding sequences constitute only a small proportion of the genome, non-protein coding RNAs (ncRNAs), which constitute > 70 % of the human genome [1], are becoming the subject of increasing attention because of their powerful regulator roles [2]. It is evident that ncRNA, like microRNAs and long ncRNAs (lncRNA) have roles in the emergence of a wide range of human diseases including cancers [3–5]. Commonly, lncRNAs are defined as ncRNAs longer than 200 nucleotides. They have multiple functions including acting as structural components, regulating protein trafficking, regulating transcription and cell metabolism, and modulating protein and RNA activity [6, 7].

Our former study using gene expression microarray revealed the non-protein coding RNAs (ncRNAs) – nuclear-enriched Abundant Transcript 1 (NEAT1) elevated in peripheral blood from patients with CRC compared with healthy participants [8].

NEAT1 is an essential component of nuclear paraspeckles [9, 10]. Paraspeckles are formed by the binding of Paraspeckle Protein (PSP) 1, PSP2 and p54^nrb^ to the NEAT1 transcriptional start site [11, 12]. Besides participating in mediate the development of corpus luteum and mammary gland [13, 14], nuclear paraspeckles have various functional significance. Especially, these nuclear bodies may serve as a “reservoir” for mRNA nuclear retention. They have also been shown to migrate to the cytoplasm to modulate cytoplasmic proteins and RNA function [12]. The NEAT1 gene encodes two transcripts: 3.7- kb NEAT1_v1 and 23- kb NEAT1_v2 [15, 16]. Although these two variants share the same transcriptional start site, NEAT1_v2 has a tRNA-like structure at its 3’ end that is processed by RNaseP cleavage rather than a poly-A tail [15]. So the two variants shared two distinct 3′-end processing mechanisms: canonical polyadenylation for NEAT1_v1 and RNase P-mediated cleavage for NEAT1_v2. NEAT1_v2 has been strongly implicated in the formation of paraspeckles [12, 15].

In the present study, we determined the diagnostic and prognostic significance and of whole blood NEAT1 in patients with colorectal cancer.

## Results

### Whole-blood NEAT1 expressions could distinguish patients with colorectal cancer from NCs

**Fig. 1:**
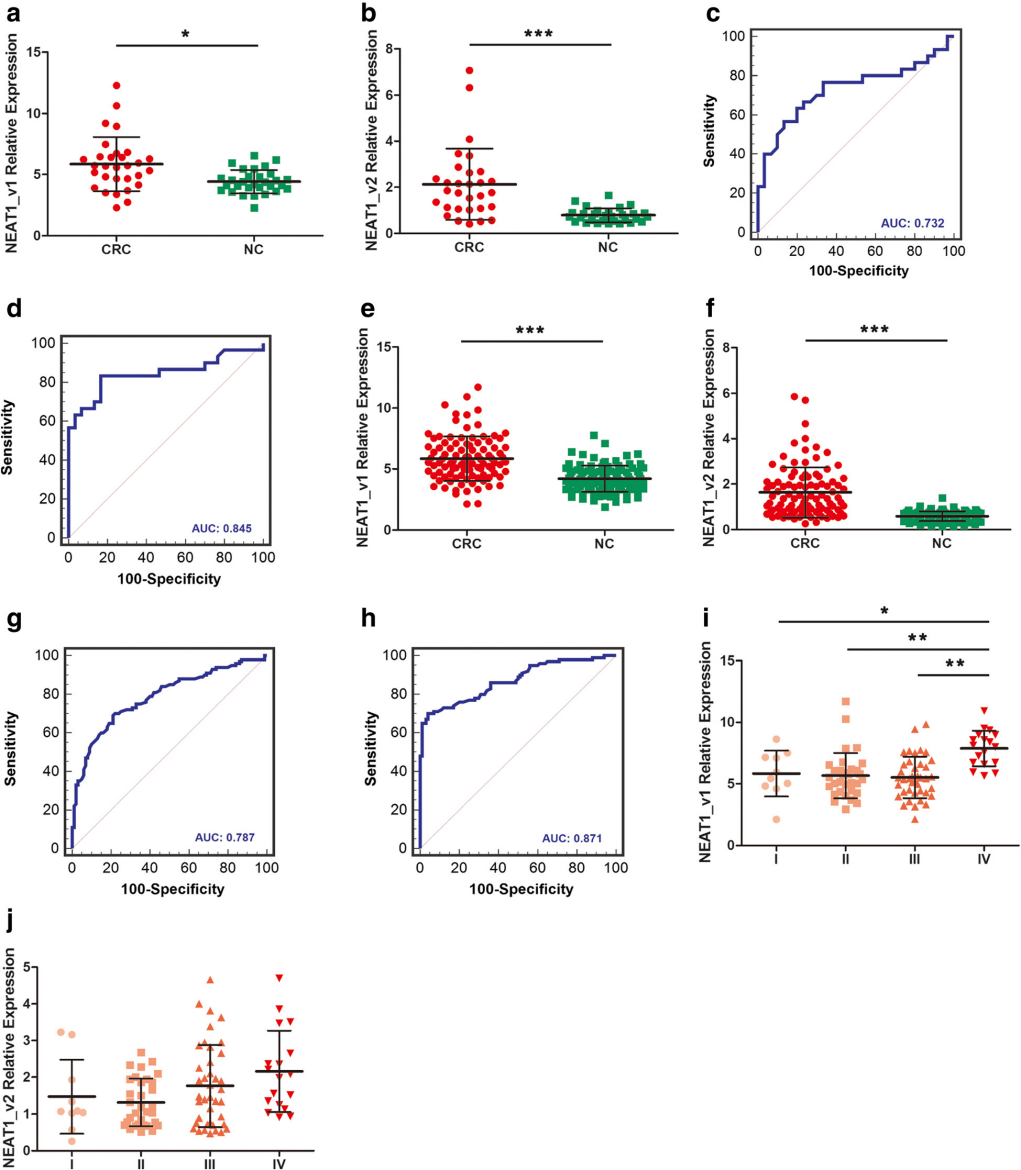
Whole blood NEAT1 expression in screening and validation phase. **a**, Plots representing different expression of NEAT1_v1 between normal controls (NC, n=30) and colorectal cancer (CRC, n=30) patients. **b**, Plots representing different expression of NEAT1_v2. **c**, Whole blood NEAT1_v1 yielded an AUC value of 0.732 (95 % CI: 0.602-0.838), with 56.7 % sensitivity and 86.7 % specificity in distinguishing colorectal cancer patients from NCs. **d**, Whole blood NEAT1_v2 yielded an AUC value of 0.845 (95% CI: 0.728-0.925), with 83.3 % sensitivity and 83.3 % specificity in distinguishing colorectal cancer patients from NCs. **e**, Plots representing different expression of NEAT1_v1 between another groups of NC (n=100) and CRC patients (n=100). **f**, Plots representing different expression of NEAT1_v2. G, ROC curve of NEAT1_v1 from another groups yielded an AUC value of 0.787 (95 % CI: 0.724-0.842), with 69.0 % sensitivity and 79.0 % specificity in distinguishing colorectal cancer patients from NCs. **h**, ROC curve of NEAT1_v2 from another groups yielded an AUC value of 0.871 (95 % CI: 0.816-0.914), with 70.0 % sensitivity and 96.0 % specificity in distinguishing colorectal cancer patients from NCs. **i**, Plots illustrating different levels of NEAT1_v1 versus TNM staging. **j**, Plots illustrating different levels of NEAT1_v2 versus TNM staging. *P<0.05, **P<0.001, ***P<0.0001

NEAT1_v1 and NEAT1_v2 levels were significantly higher in patients with colorectal cancer (*n* = 30) than in NCs (*n* = 30; *p* = 0.0021 and *p* < 0.0001, respectively; Fig. 1a-b). Moreover, ROC analysis revealed that NEAT1 expression could distinguish patients with CRC from NCs (NEAT1_v1: area under the curve [AUC] = 0.732, 95 % confidence interval [CI] = 0.602–0.838; NEAT1_v2: AUC = 0.845, 95 % CI = 0.728–0.925; Fig. 1c-d). The sensitivity and specificity of NEAT1_v1 and NEAT1_v2 in identifying colorectal cancer were 56.7 % and 86.6 %, 83.3 % and 83.3 %, respectively.

The diagnostic value of NEAT1 was validated in a larger, independent cohort of colorectal cancer patients (*n* = 100) and NCs (*n* = 100). The expression of both NEAT1 variants was significantly higher in colorectal cancer patients than in NCs (both *p* < 0.0001; Fig. 1e–f). ROC analysis confirmed the diagnostic potential of NEAT1 variants in the validation cohort. NEAT1_v1 yielded an AUC of 0.787 (95 % CI = 0.724–0.842; Fig. 1g) sensitivity of 69.0 %, and specificity of 79.0 %, and NEAT1_v2 yielded an AUC of 0.871 (95 % CI = 0.816–0.914; Fig. 1h), sensitivity of 70.0 %, and specificity of 96.0 %. Interestingly, whole blood expression of NEAT1_v1, but not NEAT1_v2, was higher in patients with stage IV colorectal cancer compared with other stages (*p* < 0.0001 and *p* = 0.132, respectively; Fig. 1i–j).

### Functional assessment of NEAT1 in colorectal cancer cells

We assessed cellular functions such as proliferation, and invasion after treatments with relevant siRNAs in two colorectal cancer cell lines. Knockdown of NEAT1_v1 could attenuated the invasive capacity, compared with cells transfected with NEAT1_v2 and control siRNA. Moreover, CCK8 assays revealed that down regulation of NEAT1_v1 lead to significant inhibition of cell growth. On the contrary, an elevated cell growth was detected when cells were treated with NEAT1_v2 siRNA (see Additional file 1).

### Validation of NEAT1 expression in tissues

To further validate that whole blood NEAT1 levels might accurately reflected the concentration in colorectal cancer tissues, we determined the relationship between NEAT1 expression in primary colorectal cancer tissues, para-tumor tissues with matched whole blood.

Twenty-nine patients from validation cohort with stage I to stage IV colorectal cancer were enrolled. However, no correlation of both NEAT1 variants was found between tissues and blood (see Additional file 2).

Notably, we found a higher content of NEAT1_v1 in patients with metastasis colorectal cancer before. So we also inferred that such higher expression might derived from metastasis tissues rather than primary tumor tissues.

**Fig. 2:**
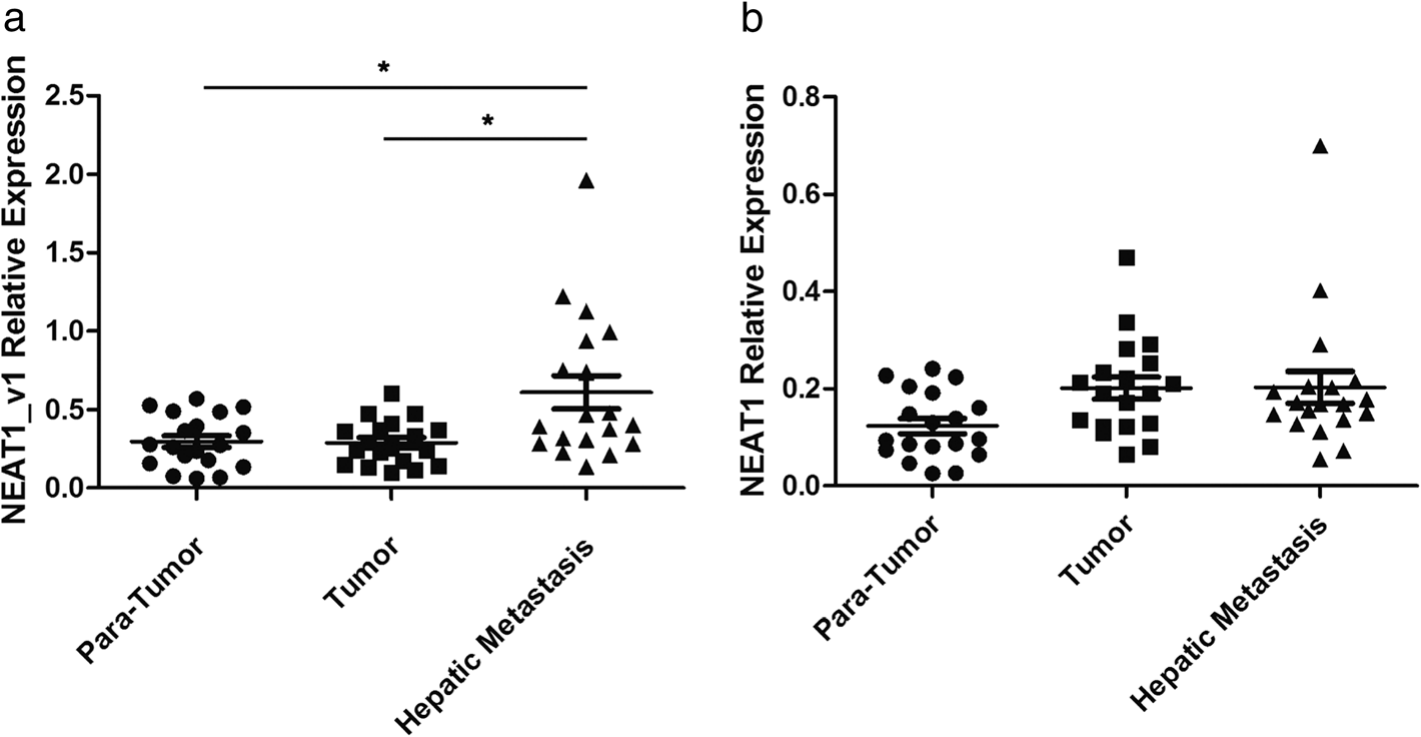
NEAT1 expression differed in the para-tumor, tumor and matched liver metastasis. (**a**) Significantly higher expression of NEAT1_v1 was detected in liver metastasis samples versus para-tumor tissues and tumor tissues (*p* = 0.003). (**b**) No significant difference was detected among primary tumor tissues , para-tumor tissues and liver metastasis (*p* = 0.076). *A two-tailed p value ≤0.05 was considered statistically significant

First of all, we examed another 19 patients who underwent simultaneously surgery for primary cancer and hepatic metastasis. Expression of NEAT1_v1 was significantly higher in metastatic hepatic tissues compared with para-tumor and primary colorectal cancer tissues (*p* = 0.003). NEAT1_v1 expression was similar between primary tumor and para-tumor tissues (*p* = 0.8214). NEAT1_v2 expression was not significantly different among para-tumor, primary tumor, and metastatic hepatic tissues (*p* = 0.076; Fig. 2a–b). These results were somewhat in accordance with what we found in whole blood. However, after comparing the expression of both NEAT1 variants in whole blood and metastasis tissues, we also found no correlation between them (see Additional file 3), which meant that there might be other reasons for such difference in whole blood rather than tissue origins.

### Neutrophil expression of NEAT1 variants was higher in colorectal cancer patients than in NCs

There was no correlation of NEAT1 expression between whole blood and tissues. Considering that immune cells comprise the majority of peripheral blood cells, we also determined NEAT1 expression from different subgroups of immune cells between colorectal cancer patients and NCs.

We separated neutrophils, monocytes, CD4-positive cells (mainly CD4-positive T helper cells), CD8-positive cells (mainly cytotoxic T cells), and CD4/CD8 double-negative cells (mainly B cells and natural killer cells) from peripheral blood (see Additional file 4).

**Fig. 3:**
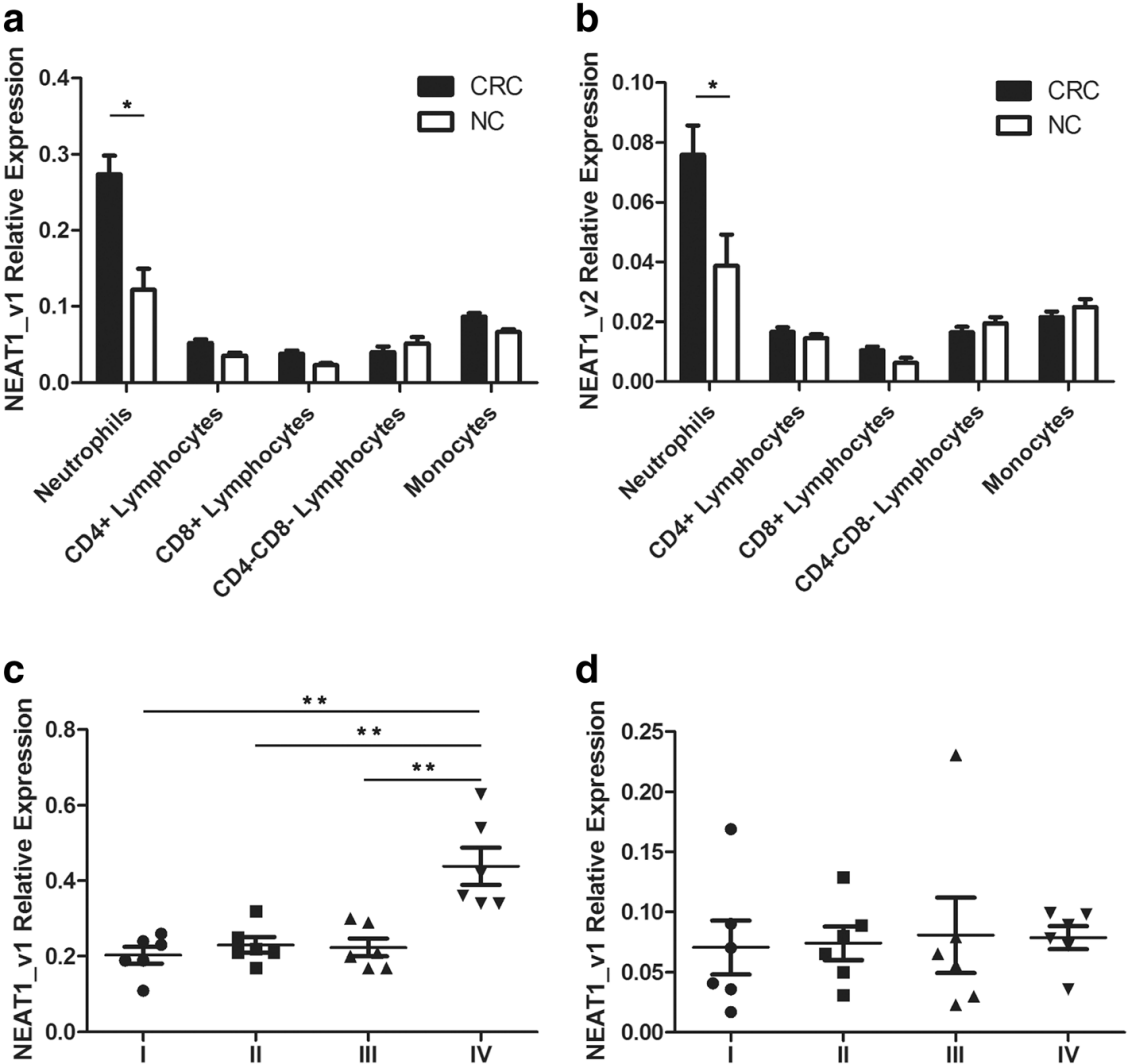
Different expression of NEAT1 isoforms in 5 groups of immune cells sorted from peripheral blood. (**a**) The NEAT1_v1 expression was different in five groups of immune cells, and varied dramatically in neutrophils from two groups of people (*p* = 0.018). (**b**) The NEAT1_v2 expression in CRC patients was also higher than that in normal controls (*p* = 0.0057). All graphs showed mean values ± SEM. *p < 0.05. (**c**) NEAT1_v1 was higher in neutrophils fro stage IV colorectal cancer patients. (**d**) No significant difference was detected in NEAT1_v2 expression in different stage

Neutrophils had the highest expression of both NEAT1 variants among the immune cell types. Expression of both variants in neutrophils were higher in colorectal cancer patients than in NCs (NEAT1_v1, *p* = 0.001; NEAT1_v2, *p* = 0.015; Fig. 3a–b). Moreover, when comparing neutrophils’ expression in different cancer stages, an elevated content of NEAT1_v1 but not NEAT1_v2 was found in stage IV patients, which was in accordance with what we found in whole blood (NEAT1_v1 Stage IV, *p* < 0.001, Fig. 3c–d). Overall, we testified that NEAT1 expression in peripheral neutrophils might be the key point in distinguishing CRC patients from healthy people.

### Whole blood expression of NEAT1 was significantly correlated with overall survival in patients with colorectal cancer

**Fig. 4:**
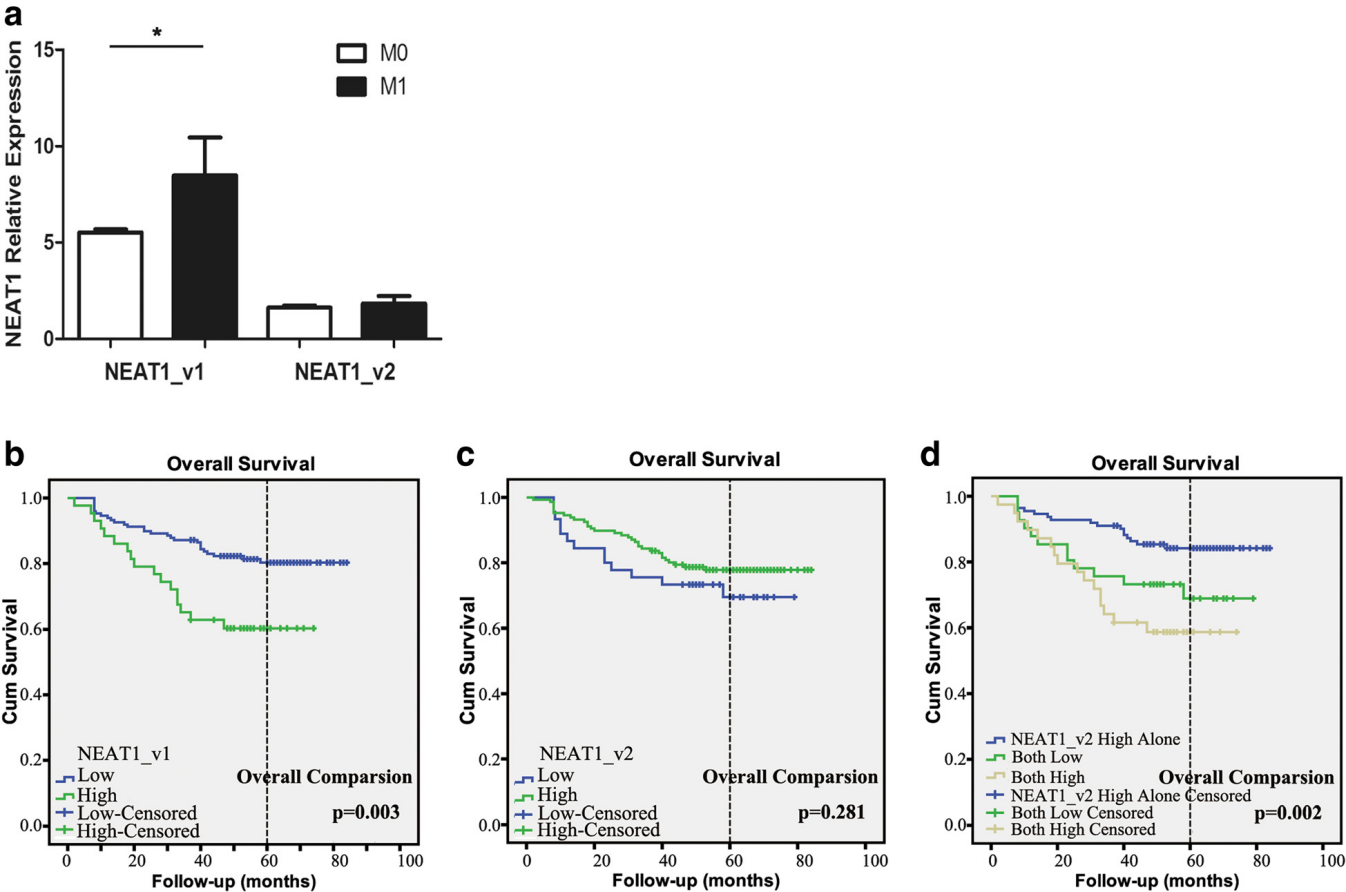
Whole blood NEAT1 expression patients with metastasis and its correlation with overall survival. (**a**) NEAT1_v1 but not NEAT1_v2 was higher in patients’ blood who had metastasis (*p* = 0.023). (**b**) Patients with higher expression of NEAT1_v1 tended to have worse overall survival (*p* = 0.003). (**c**) Compared to NEAT1_v1, no significant difference was found in NEAT1_v2 to overall survival. (**d**) Higher expression of NEAT1_v2 alone (higher expression of NEAT1_v2 without higher expression of NEAT1_v1) had better overall survival than that of both low expressions (*p* = 0.036) and both high expressions (*p* < 0.001). OS between patients from both low expressions and both high expressions had no significant difference (*p* = 0.303).*A two-tailed p value ≤0.05 was considered statistically significant

After proving the diagnostic value of whole blood expression of NEAT1, we proceeded to verify its function in prognosis. We determined the correlation of whole blood NEAT1_v1 and NEAT1_v2 expression with clinical outcomes in 191 colorectal cancer patients with available clinicopathological data. NEAT1_v1 were significantly higher in colorectal cancer patients with distant metastases (M1) than in those without metastasis (M0; *p* = 0.023), whereas NEAT1_v2 levels did not differ between the M1 and M0 groups (*p* = 0.9917; Table 1 and Fig. 4a).

**Table 1:** Relationship between NEAT1 and clinical features of patients with colon cancer in the whole blood

Clinical variables	Classification	N	NEAT1_v1		NEAT1_v2	
			Mean	*P* value	Mean	*P* value
Gender	Male	101	6.26	0.098	1.65	0.987
	Female	90	5.38		1.65	
Age (years)^a^	<56	100	5.60	0.253	1.62	0.769
	≥56	91	6.21		1.69	
Tumor (T) stage	T1–2	26	6.16	0.287	1.46	0.476
	T3–4	165	5.80		1.68	
Nodal (N) status	N0	95	5.45	0.287	1.54	0.464
	N1	48	6.02		1.65	
	N2	48	6.45		1.87	
Distant metastasis (M)	M0	170	5.52	0.023*	1.63	0.532
	M1	21	8.49		1.84	
AJCC stage^b^	I–II	94	5.45	0.144	1.55	0.374
	III–IV	97	6.23		1.74	
Lymphovascular invasion	No	141	5.55	0.497	1.70	0.463
	Yes	50	6.67		1.52	
Perineural invasion	No	163	5.55	0.549	1.67	0.701
	Yes	28	7.54		1.55	
Extranodal tumor deposits	No	163	5.53	0.139	1.66	0.800
	Yes	28	7.66		1.58	
Differentiation	G1–G2	149	5.92	0.587	1.64	0.880
	G3–G4	42	5.57		1.68	
Pathology	Adenocarcinoma	164	5.93	0.412	1.68	0.465
	Mucinous or signet-ring carcinoma	27	5.30		1.46	
Maximum size (cm)^a^	<5	118	6.02	0.344	1.67	0.687
	>5	73	5.48		1.58	

*A two-tailed *p* value ≤0.05 was considered statistically significant

^a^The mean age and tumor size, respectively

^b^Abbreviations: AJCC, American Joint Committee on Cancer (AJCC)

The median follow-up duration was 56 (2–83) months. Patients with high NEAT1_v1 expression had shorter OS and median survival times than patients with low NEAT1_v1 expression (*p* = 0.003; Fig. 4b).

Univariate analysis revealed that nodal status (N0/N1 *p* < 0.001; N0/N2 *p* = 0.001), distant metastasis (*p* < 0.001), lymphatic/vascular invasion (*p* < 0.001), perineural invasion (*p* = 0.001), extranodal tumor deposit (*p* = 0.005), American Joint Committee on Cancer stage (*p* < 0.001), and NEAT1_v1 expression (*p* = 0.004) were significantly associated with OS. However, in the multivariate analysis, only nodal status (N0/N1 *p* < 0.001; N0/N2 *p* = 0.017) and distant metastasis (*p* < 0.001) remained significantly independent prognostic factors for OS (Table 2). NEAT1_v1 expression was found to have limited prognostic value for OS (*p* = 0.068).

**Table 2:** Cox analysis of the prognostic variables on the overall survival in patients ( Whole blood)

Prognosis variables	Overall survival	
	*P* value	HR (95 % CI)
Univariate analysis		
NEAT1_v1, Low/High	0.004*	0.415 (0.227–0.758)
NEAT1_v2, Low/High	0.285	1.421 (0.746–2.708)
NEAT1_v2, High Alone^a^	0.006*	0.426 (0.231–0.784)
Gender, Male/Female	0.399	1.290 (0.714–2.330)
Age, <56/≥56 years^b^	0.967	0.988 (0.544–1.793)
Tumor (T) stage, T1–2/T3–4	0.124	0.399 (0.124–1.288)
Nodal (N) status, N0/N1	<0.001*	0.120 (0.056–0.257)
N0/N2	<0.001*	0.297 (0.143–0.617)
Distant metastasis (M), M0/M1	<0.001*	0.102 (0.055–0.188)
Lymphatic/vascular invasion, No/Yes	<0.001*	0.243 (0.135–0.438)
Perineural invasion, No/Yes	0.001*	0.342 (0.179–0.653)
Extranodal tumor deposit, No/Yes	0.005*	0.389 (0.201–0.753)
AJCC Stage^c^, I–II/III–IV	<0.001*	0.205 (0.066–0.426)
Differentiation, G1–G2/G3–G4	0.058	0.543 (0.289–1.022)
Pathology, Adenocarcinoma/Mucinous or signet-ring carcinoma	0.712	0.859 (0.384–1.923)
Size, <5/≥5^b^	0.598	1.187 (0.627–2.247)
Multivariate analysis		
NEAT1_v1, Low/High	0.068	
NEAT1_v2, High Alone^a^	0.038*	0.519 (0.280–0.965)
Nodal (N) status, N0/N1	<0.001*	0.199 (0.088–0.452)
N0/N2	0.017*	0.402 (0.189–0.851)
Distant metastasis (M), M0/M1	<0.001*	0.193 (0.099–0.374)
Lymphatic/vascular invasion, No/Yes	0.996	
Perineural invasion, No/Yes	0.366	
Extranodal tumor deposit, No/Yes	0.645	
AJCC Stage^c^, I–II/III–IV	0.498	

*A two-tailed *p* value ≤0.05 was considered statistically significant

^a^High expression of NEAT1_v2 with low expression of NEAT1_v1

^b^The mean age and tumor size, respectively

^c^Abbreviations: AJCC, American Joint Committee on Cancer (AJCC)

Although OS did not differ in patients with different levels of NEAT1_v2 (*p* = 0.281, Fig. 4c), higher expression of NEAT1_v2 without higher expression of NEAT1_v1 (high expression of NEAT1_v2 alone) represented better OS compared with low and high expression of both variants (*p* = 0.036 and *p* < 0.001, respectively) (Fig. 4d). There was no significant difference in OS between patients with low expression of both variants compared with those with high expression of both variants (*p* = 0.303). Multivariate analysis, revealed that high expression of NEAT1_v2 alone could be an independent prognostic factor for improved OS (*p* = 0.0038). Moreover, we found that higher expression of NEAT1_v1 was almost combined with higher expression of NEAT1_v2 (107/111 patients).

## Discussion

Colorectal cancer is one of the most common malignancies worldwide. It is the third common cancer and the fifth leading cause of death in China [17, 18]. Early diagnosis of colorectal cancer is important for improving treatment response, patients’ survival, and quality of life. Blood-based tests to detect tumor markers, such as carcinoembryonic antigen, may help to evaluate disease status, but their accuracy and efficacy are controversial [19, 20]. Ideally, diagnostic tests for the early detection of colorectal cancer should be simple, accurate, and minimally invasive. Considerable studies have identified and developed novel biomarkers for diagnosis, prognosis, and predicting treatment response [21–24]. Using independent patient cohorts, we demonstrated for the first time that whole blood NEAT1_v1 and NEAT1_v2 may be valuable diagnostic biomarkers in colorectal cancer. However, NEAT1_v2 seemed to be a more sensitive and specific biomarker compared with NEAT1_v1 (70 % vs. 69.0 % and 96.0 % vs. 79.0 %, respectively).

Our study also demonstrated the prognostic potential of NEAT1 expression in colorectal cancer. Whole blood NEAT1_v1 expression was higher in patients with distant metastasis, and patients with high NEAT1_v1 expression had poorer OS. However, NEAT1_v1 expression was found not to be an independent prognostic factor for OS in the multivariate analysis. Furthermore, NEAT1_v1 was higher in hepatic metastatic tissues than in primary colorectal cancer tissues, suggesting that elevated NEAT1_v1 expression in whole blood may originate from metastatic tissues. Further studies are needed to assess the potential contribution of other metastatic sites such as the lung and bone to NEAT1_v1 expression in the peripheral whole blood of colorectal cancer patients.

Interestingly, despite sharing the same transcriptional start site, NEAT1_v1 and NEAT1_v2 appeared to have different roles in predicting clinical outcomes. We found that high NEAT1_v2 combined with low NEAT1_v1 expression was associated with improved OS in colorectal cancer patients. Furthermore, NEAT1_v2 expression was an independent prognostic factor for OS in the multivariate analysis. In contrast, elevated NEAT1_v1 was associated with poor OS because of its association with metastasis. We also found that patients with simultaneous low or high expression of NEAT1_v1 and NEAT1_v2 had poor OS. Based on these findings, we believe that NEAT1_v2 may play a protective role in colorectal cancer. In patients with low expression of both variants, NEAT1_v2 is unable to perform its protective function. On the other hand, in patients with high expression of both variants, the putative protective role of NEAT1_v2 may be masked by high NEAT1_v1 expression, resulting in poor OS and tendency toward metastasis. Although mechanisms underlying this dynamic equilibrium may be difficult to investigate, it is clear that the differential expression of NEAT1 variants in whole blood has prognostic value in colorectal cancer.

The ratio of the two variants of NEAT1 is regulated by differential use of polyadenylation signals. Naganuma et al. demonstrated that one of the paraspeckle proteins (PSPs), HNRNPK competed with CPSF6 (CFIm68) for binding to NUDT21 (CFIm25), leading to the accumulation of NEAT1_v2 [25]. Interestingly, Chioniso et al. indenified CFIm25 as a tumour suppresser as RNA 3′-end-processing factor in glioblastoma [26]. So further research on the polyadenylation signals in NEAT1 edition and its function in colorectal cancer is required. Furthermore, other roles of NEAT1 in mediating tumorigenesis and metastasis has recently received increasing attention [27–29]. Choudhry et al. found that NEAT1 was regulated by hypoxia-inducible factor-2 and hypoxia-induced NEAT1 accelerates cellular proliferation, improves cell survival, and reduces apoptosis (28). Chakravarty et al. reported that androgen receptor antagonist-resistant prostate cancer cells express high levels of NEAT1, which was shown to drive their oncogenic growth (29). In the present study, we demonstrated that NEAT1_v1 could improve tumor cells growth and invasion, while NEAT1_v2 had reverse function in proliferation, which was somewhat in corresponding with the prognostic potential of whole blood NEAT1 expression. Moreover, we found that NEAT1_v1 was highly expressed in metastatic hepatic tissues from colorectal cancer patients. These findings suggest that NEAT1 has both pro-oncogenic and pro-metastatic effects in colorectal cancer. A recently study demonstrated that NEAT1 and another lncRNA, metastasis-associated lung adenocarcinoma transcript 1 (MALAT1) colocalize to hundreds of genomic sites and may have complementary functions [30]. MALAT1, an 8.1 kb lncRNA , is overexpressed in many malignant tumors, including human primary colorectal cancer. MALAT1 upregulation has been reported to promote colorectal cancer development via its target protein A-kinase anchor protein [31]. Together, these studies indicate that NEAT1 has a potentially complex role in driving tumorigenesis.

In the present study, we also investigated the possible sources contributing to the differential whole blood expression of NEAT1 between colorectal cancer patients and NCs. The expression of NEAT1 was higher in patients’ blood than tissues. We detected higher expression in metastasis liver tissues and proved its mechanism in tumor cells proliferation and invasion. The in vitro experiments enclosed a contradictory mechanism of NEAT1 variants in tumor development and invasion, which was in accord with the prognostic potential in whole blood. NEAT1_v1 acted as a carcinogenic factor, while NEAT1_v2 was a protective one. However, fact that no correlation was found between whole blood and tissues could not be ignored. One of the possible explanation might be the degradation of NEAT1 in whole blood which derived from tissues. Moreover, expression of NEAT1 was higher in whole blood than in tissues. This suggests that cancer-related sources do not fully account for the increased expression in whole blood. Our findings indicate that neutrophils may mostly contribute to the elevated expression of NEAT1 in whole blood, as both NEAT1_v1 and NEAT1_v2 expression were higher in neutrophils from colorectal cancer patients than in those from NCs, and a higher NEAT1_v1 expression was also detected in neutrophils from stage IV patients, which was in correspondence with the difference found in whole blood.

The functional roles of peripheral blood and tumor-associated neutrophils have been investigated in cancer patients. Clinical research has primarily evaluated the prognostic role of peripheral neutrophils in cancer patients [32–34]. These studies have shown that a high neutrophil to lymphocyte ratio is an independent prognostic factor for survival. On the other hand, fundamental research has mostly focused on tumor-associated neutrophils [35] and their migration [36–38], proliferation, correlation with other immune cells such as myeloid-derived suppressor cells [39–41], and paradoxical functions on tumorgenesis [42–45]. Therefore, the functional roles of peripheral neutrophils in tumorigenesis are still unclear. Although the role of NEAT1 in immune system function, especially during viral infection (e.g., human immunodeficiency viral infection), has been intensely investigated [46–48], its function of NEAT1 in neutrophils has not been fully addressed. Considering that NEAT1 acts as one of the main structural components in paraspeckles and possess a wide range of known or unknown function, its role in peripheral neutrophils of colorectal cancer patients is warranted. Whether increased NEAT1 expression in neutrophils is a cause or by-product of tumorigenesis and whether there are other potential sources of NEAT1 are still unclear.

Our findings indicated the significant promise of whole blood NEAT1 expression as a diagnostic marker in colorectal cancer and thus, warrants confirmation in larger studies. These studies should include patients in the same general condition and use a uniform method to detect RNA expression to help eliminate any potential coincidental bias. Differential expression of NEAT1 variants may be potentially relevant in other cancer types; therefore, further studies in patients with other cancer types are also needed. Moreover, despite no correlation of NEAT1 expression between whole blood and tissues was found, an elevated expression in metastasis was certainty. So basic research on NEAT1 in carcinogenesis is indispensable and is in process. Moreover, higher NEAT1 expression in CRC patients’ whole blood did not correlated with tissues and it might originated from its different content in neutrophils. In summary, we believe that such difference in whole blood derived from numerous sources, but mainly from neutrophils. However, the function of NEAT1 on neutrophils needs to be uncovered in the future.

In conclusion, our study established and validated NEAT1 as a new diagnostic biomarker in colorectal cancer. In addition, NEAT1_v1 and NEAT1_v2 expression were associated with different prognostic outcomes. Elevated whole blood NEAT1 expression may derive, in part, from metastatic tissues and neutrophils. Further research is needed to fully clarify the clinical significance of high neutrophil NEAT1 expression in colorectal cancer patients.

## Materials and Methods

### Study design and patients

The study design and patient characteristics are confirmed (see Additional files 5 and 6). Study participants were recruited from the Fudan University Shanghai Cancer Center (China) between 2006 and 2012. Patients who underwent any preoperative radiotherapy or chemotherapy before blood collection, and those with inflammatory bowel disease, hereditary colorectal cancer, or other rare tumor types were excluded. Healthy volunteers with normal colonoscopy results for colorectal cancer served as the normal controls (NCs). The study was approved by the Medical Ethics Committee of Fudan University Shanghai Cancer Center, and written informed consent was obtained from all participants. TNM classification and differentiation grade were determined according to the 2010 American Joint Committee on Cancer criteria (7th edition). Clinicopathological data were obtained retrospectively from the patients’ electronic medical records.

The diagnostic value of whole blood NEAT1 was initially evaluated in a test cohort comprised of 30 colorectal cancer patients and 30 NCs and then validated in a large, independent cohort of 100 patients and 100 NCs. Tumor tissues, para-tumor tissues of 29 patients (stage I to stage IV) from validation cohort were collected and compared with matched blood sample. Moreover, another 19 patients receiving simultaneously resection of primary tumor tissues and liver metastasis were also enrolled to investigate the correlation of NEAT1 between whole blood and tissues.

Subsequently, a survival analysis was performed in an additional 191 patients with available clinicopathological and prognostic data. Finally, to identify the origin of whole blood NEAT1 expression, NEAT1 expression in primary colorectal cancer and matched hepatic metastatic tissues and immune cells was determined. Primary colorectal cancer and matched hepatic metastatic tissues were obtained from 19 colorectal cancer patients who had undergone simultaneous resection of colorectal cancer and hepatic metastasis. Immune cells were isolated from peripheral blood samples obtained from 12 colorectal cancer patients and 12 NCs.

### Blood and tissue collection and immune cell isolation

All blood samples were obtained prior to surgery or adjuvant therapy. To investigate NEAT1 expression for clinical outcomes, peripheral blood (2.5 mL) samples were collected into PAXgene Blood RNA Tubes (PreAnalytiX). Normal and cancerous colorectal tissues and matched hepatic metastatic tissues were collected immediately after surgery, washed in cold PBS, and stored in RNAlater RNA Stabilization Reagent (Qiagen) at −20 °C for future use. For immune cell sorting, peripheral blood samples (20 mL) were collected and stored in EDTA anticoagulant tubes. All immune cells were isolated < 12 h after collection. Neutrophils were isolated using improved Ficoll Solution (TBD). CD4-positive and CD8-positive T cells were isolated from peripheral blood mononuclear cells by fluorescence-activated cell sorting (Aria II Cell Sorter; BD Biosciences) with fluorescein isothiocyanate-conjugated anti-CD4 and allophyocyanin-conjugated anti-CD8 monoclonal antibodies (BD Biosciences) according to manufacturer’s instructions. All fluorescence-activated cell sorting data were analyzed using FlowJo software (Tree Star).

### RNA isolation and quantitative real-time reverse transcription-PCR

Total RNA was extracted from peripheral blood samples using the PAXgene Blood RNA System (PreAnalytiX). Total RNA was isolated from immune cells and tissues using TRIzol Reagent (Sigma) according to the manufacturer’s instructions. RNA was quantified using a NanoDrop spectrophotometer. For each sample, RNA (320 ng) was reverse transcribed using the QuantiTect Reverse Transcription Kit (Qiagen). cDNA was amplified using SYBR Premix DimerEraser (Perfect Real Time; Takara Biotechnology), and real-time quantification was performed using the Applied Biosystems 7900HT Fast Real-Time PCR System (Life Technologies). As we had previously identified DECR1 (2,4-dienoyl-coenzyme A reductase 1 mitochondrial) as a stably expressed reference gene in whole blood samples [8, 49, 50], it was used as a reference gene for the blood sample analysis. β-actin was used as a reference gene for the tissue sample analysis. Primer sequences are provided in Additional file 7. All real-time PCR products were verified by DNA sequencing. Relative gene expression levels were estimated using the comparative Ct method of relative quantification, normalizing the Ct values relative to the reference gene. Relative gene expression is presented as 2^-∆Ct^, where ∆Ct = Ct _colorectal cancer_ –Ct _Control_.

### Cell lines and cell culture conditions

Two human colorectal cancer cell lines (HCT116, LOVO) were obtained from the Type Culture Collection of the Chinese Academy of Sciences (Shanghai, China) and were cultured in McCoy’s 5a and F-12 K Medium respectively. All medium contained 10 % fetal bovine serum (FBS). Medium and FBS were obtained from GIBCO® (Life Technologies, USA).

### Invasion and proliferation assays

NEAT1 is located in cell nucleus and is hard to knockdown with simple siRNA stably. So we used a mixed siRNA pool to silence NEAT1 variants respectively. In transwell assay, transfected cells (3 × 10^5^cells/well) were seeded in serum-free medium in 12-well Transwell Chamber (BD Biosciences, USA). 50ul diluted Matrigel was plated in chambers. They were placed in cell culture plates containing 10 % FBS and incubated for 48 h. After that, membranes were washed, fixed and stained with Crystal Violet. Invading cells in 10 microscopic fields were counted.

Cell Counting Kit 8 (CCK-8, Donjindo) was used to assessed cell viability. Treated cells were seeded into 96-well plates at an initial density of 1 × 10^3^cells/well. After 24, 48, 72, and 96 h of cultivation, CCK-8 solution was added to each well and incubated for 2 h. The absorbance was measured by scanning with a microplate reader (MRX; Dynex Technologies, West Sussex, United Kingdom) at 450 nm.

Each independent experiment was performed 3 times. Results are presented as mean ± SE.

### Statistical analyses

Statistical analyses were performed using SPSS v.19.0 (IBM Corp.). Receiver operating characteristic (ROC) analysis was performed to determine the ability of whole blood NEAT1 expression to distinguish colorectal cancer patients from NCs. The Kolmogorov-Smirnov test was used to verify the normal distribution of cardinal variables. Exploratory comparison of normally distributed and non-normally distributed independent groups was performed using t-tests and Mann–Whitney U (2 groups) or Kruskal-Wallis tests (>2 groups), respectively. The Friedman test was used to analyze differences among 3 paired samples. Pearson analysis was performed to explore the correlation of NEAT1 between blood and tissues. Overall survival (OS) was estimated using Kaplan–Meier curves and compared using the log-rank test. OS was calculated as the time from date of definitive diagnosis to date of death by any cause. Prognostic factors were determined using Cox regression analysis. A two-tailed *p* value < 0.05 was considered statistically significant.

### Ethics, consent and permissions

Written informed consent was obtained from all participants. Study was approved by the Medical Ethics Committee of Fudan University Shanghai Cancer Center (reference number 050432-4-1212B).

### Consent to publish

We have obtained consent from all participants to report data to publish.

## Additional files

Additional file 1: Figure S1. Functional assays of NEAT1 on colorectal cancer cells. Knockdown of NEAT1_v1 expression suppressed cell invasion and proliferation, while knockdown of NEAT1_v2 promote cell proliferation. (PDF 395 kb) Additional file 2: Table S1. Correlation of NEAT1 expression between blood and matched tissues. Blood and matched tissues were collected from 46 patients (Stage I-IV). Correlation analysis was performed and *p*-value was present in the table. (PDF 303 kb) Additional file 3: Table S2. Correlation of NEAT1 expression between blood and matched tissues in patients with simultaneously surgery for primary cancer and hepatic metastasis. Blood and matched tissues were collected from 19 patients with simultaneously surgery. Correlation analysis was performed and *p*-value was present in the table. (PDF 151 kb) Additional file 4: Figure S2. 5 groups of immune cells separated from peripheral blood from patients and healthy donors. Neutrophils were isolated using improved Ficoll Solution. Monocytes, CD4^+^Lymphocytes, CD8^+^ Lymphocytes, and CD4^−^CD8^−^ Lymphocytes were isolated from peripheral blood mononuclear cells by fluorescence-activated cell sorting with anti-CD4 and anti-CD8 monoclonal antibodies. (PDF 85.8 kb) Additional file 5: Figure S3. Study design. The blood gene expression was identified using a screening set and an independent validation set. Blood from another 191 patients was detected for clinical outcomes. Primary lesion and metastasis with matched blood were also investigated. Another group of blood were detected on immune cells. (PDF 87.5 kb) Additional file 6: Table S3. Characteristics of the CRC and control populations. (PDF 183 kb) Additional file 7: Table S4. Primer sequences for NEAT1 and reference genes. (PDF 132 kb)

## Abbreviations

NEAT1: Nuclear-enriched abundant transcript 1
CRC: Colorectal cancer
PSP: Paraspeckle protein
lncRNA: Long non-coding RNA
ROC: Receiver operating characteristic curve
AUC: Area under the curve
OS: Overall survival
NC: Normal control
AJCC: American joint committee on cancer
TAN: Tumor associated neutrophil
DECR1: 2,4-dienoyl-coenzyme A reductase 1 mitochondrial
MALAT1: Metastasis-associated lung adenocarcinoma transcript 1
