# Supplementary material for: Nuclear-enriched abundant transcript 1 as a diagnostic and prognostic biomarker in colorectal cancer
Source: Mol Cancer. 2015 Nov 9;14:191. doi: 10.1186/s12943-015-0455-5 (PMC4640217; doi:10.1186/s12943-015-0455-5)
Supplement: Additional file 1: Figure S1. — Functional assays of NEAT1 on colorectal cancer cells. Knockdown of NEAT1_v1 expression suppressed cell invasion and proliferation, while knockdown of NEAT1_v2 promote cell proliferation. (PDF 395 kb) [file 12943_2015_455_MOESM1_ESM.pdf]

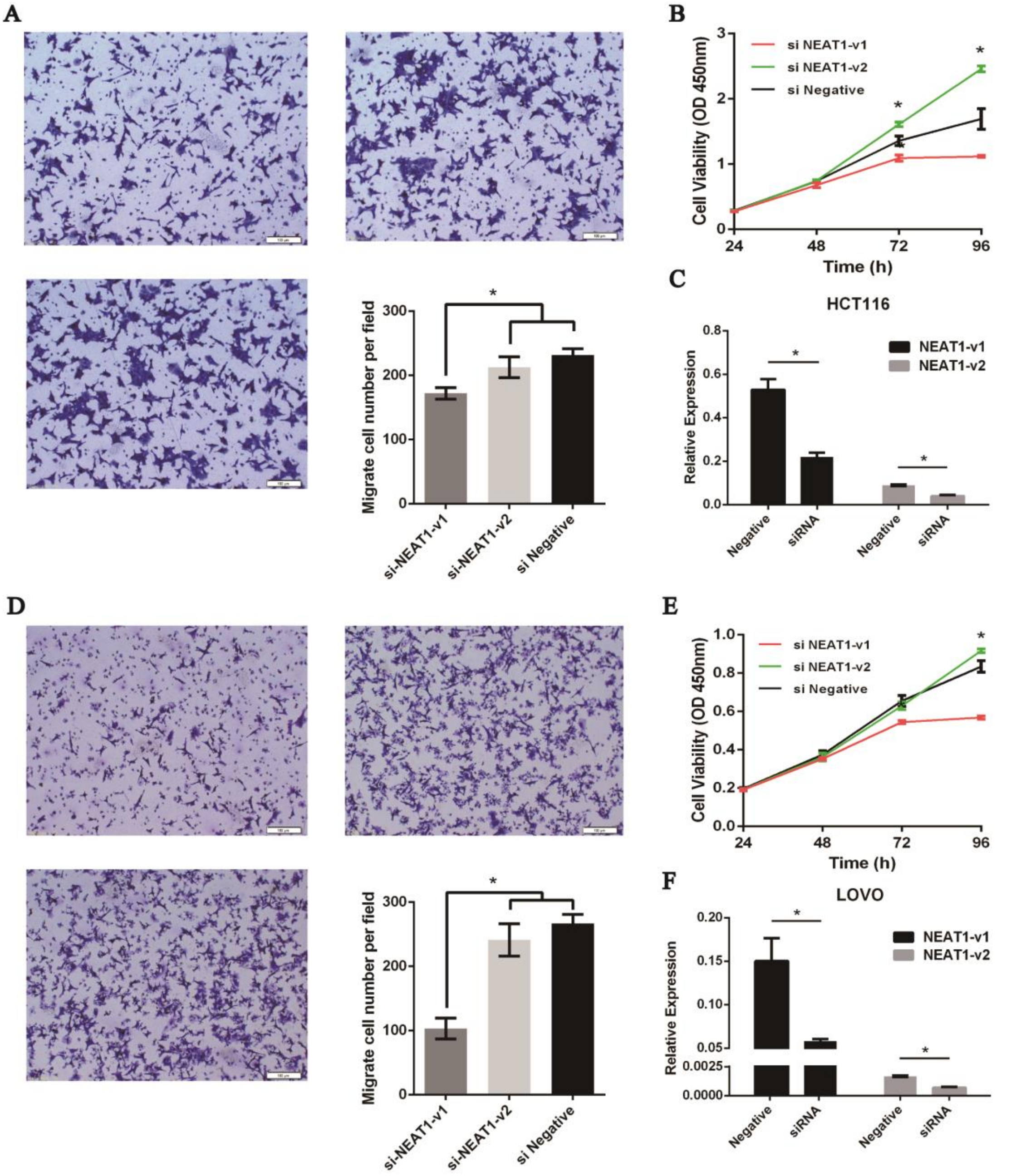

Figure S1. Functional assays of NEAT1 on colorectal cancer cells. A and D, Transwell invasion assay demonstrated weaken invasive capacity of HCT116 and LOVO cell lines after NEAT1_v1 knockdown. Images of cells were taken at 100μm. B and E, CCK8 assays demonstrated that cell growth of HCT116 and LOVO was significantly inhibited when treated with NEAT1_v1 siRNA at 72 to 96 hours and 96 hours respectively, compared with controls. Reverse result was detected when cell lines were treated with NEAT1_v2 siRNA. C and F, All knockdown efficiency was confirmed in cell lines. * P<0.05
