# Supplementary material for: Nuclear-enriched abundant transcript 1 as a diagnostic and prognostic biomarker in colorectal cancer
Source: Mol Cancer. 2015 Nov 9;14:191. doi: 10.1186/s12943-015-0455-5 (PMC4640217; doi:10.1186/s12943-015-0455-5)
Supplement: Additional file 2: Table S1. — Correlation of NEAT1 expression between blood and matched tissues. Blood and matched tissues were collected from 46 patients (Stage I-IV). Correlation analysis was performed and p-value was present in the table. (PDF 303 kb) [file 12943_2015_455_MOESM2_ESM.pdf]

Table S1. Correlation of NEAT1 expression between blood and matched tissues.

Correlation of NEAT1 expression between blood and matched tissues					
		**Tumor Tissues**		**Para-Tumor Tissues**	
		**NEAT1_v1**	**NEAT1_v2**	**NEAT1_v1**	**NEAT1_v2**
**Blood**	**p value**	0.733	0.475	0.764	0.771
	**Pearson correlation**	0.066	-0.138	0.058	0.057

Blood and matched tissues were collected from 29 patients (Stage I-IV). Correlation analysis was performed. Pearson correlation and p-value was present in the table.
