# Supplementary material for: Nuclear-enriched abundant transcript 1 as a diagnostic and prognostic biomarker in colorectal cancer
Source: Mol Cancer. 2015 Nov 9;14:191. doi: 10.1186/s12943-015-0455-5 (PMC4640217; doi:10.1186/s12943-015-0455-5)
Supplement: Additional file 3: Table S2. — Correlation of NEAT1 expression between blood and matched tissues in patients with simultaneously surgery for primary cancer and hepatic metastasis. Blood and matched tissues were collected from 19 patients with simultaneously surgery. Correlation analysis was performed and p-value was present in the table. (PDF 151 kb) [file 12943_2015_455_MOESM3_ESM.pdf]

Table S2. Correlation of NEAT1 expression between blood and matched tissues in patients with simultaneously surgery for primary cancer and hepatic metastasis

Correlation of NEAT1 expression between blood and matched tissues							
		**Tumor Tissues**		**Para-Tumor Tissues**		**Hepatic Meatstasis**	
		**NEAT1_v1**	**NEAT1_v2**	**NEAT1_v1**	**NEAT1_v2**	**NEAT1_v1**	**NEAT1_v2**
**Blood**	**p value**	0.998	0.904	0.643	0.206	0.656	0.901
	**Pearson Correlation**	-0.001	0.044	0.168	-0.437	0.161	0.045

Blood and matched tissues were collected from 19 patients with simultaneously surgery. Correlation analysis was performed. Pearson correlation and p-value was present in the table.
