# Supplementary material for: Nuclear-enriched abundant transcript 1 as a diagnostic and prognostic biomarker in colorectal cancer
Source: Mol Cancer. 2015 Nov 9;14:191. doi: 10.1186/s12943-015-0455-5 (PMC4640217; doi:10.1186/s12943-015-0455-5)
Supplement: Additional file 4: Figure S2. — 5 groups of immune cells separated from peripheral blood from patients and healthy donors. Neutrophils were isolated using improved Ficoll Solution. Monocytes, CD4+Lymphocytes, CD8+ Lymphocytes, and CD4−CD8− Lymphocytes were isolated from peripheral blood mononuclear cells by fluorescence-activated cell sorting with anti-CD4 and anti-CD8 monoclonal antibodies. (PDF 85.8 kb) [file 12943_2015_455_MOESM4_ESM.pdf]

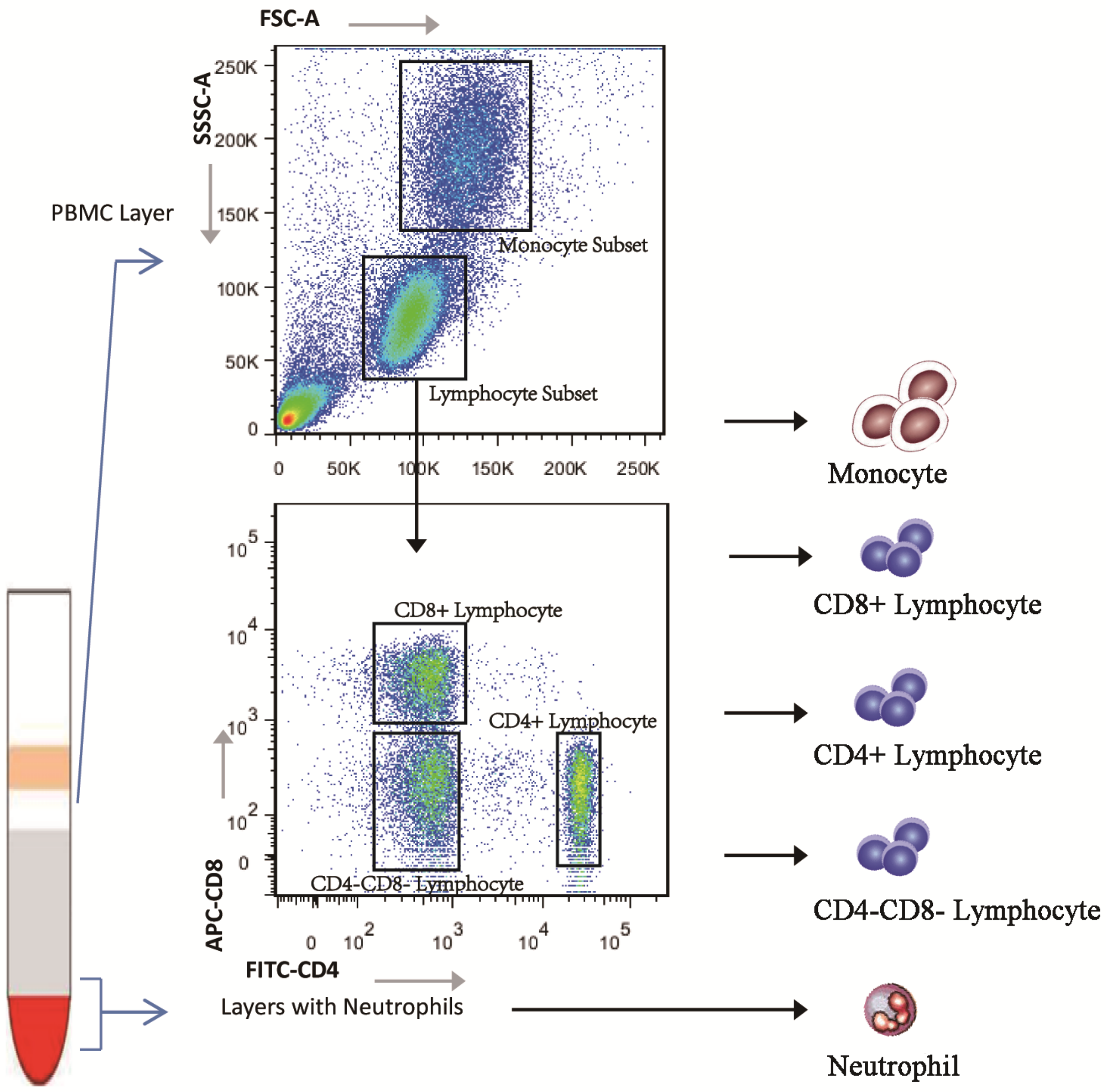

Figure S2. 5 groups of immune cells separated from peripheral blood from patients and healthy donors. Neutrophils were isolated using improved Ficoll Solution. Monocytes, CD4+Lymphocytes, CD8+ Lymphocytes, and CD4-CD8- Lymphocytes were isolated from peripheral blood mononuclear cells by fluorescence-activated cell sorting with anti-CD4 and anti-CD8 monoclonal antibodies.
