# Supplementary material for: Nuclear-enriched abundant transcript 1 as a diagnostic and prognostic biomarker in colorectal cancer
Source: Mol Cancer. 2015 Nov 9;14:191. doi: 10.1186/s12943-015-0455-5 (PMC4640217; doi:10.1186/s12943-015-0455-5)
Supplement: Additional file 5: Figure S3. — Study design. The blood gene expression was identified using a screening set and an independent validation set. Blood from another 191 patients was detected for clinical outcomes. Primary lesion and metastasis with matched blood were also investigated. Another group of blood were detected on immune cells. (PDF 87.5 kb) [file 12943_2015_455_MOESM5_ESM.pdf]

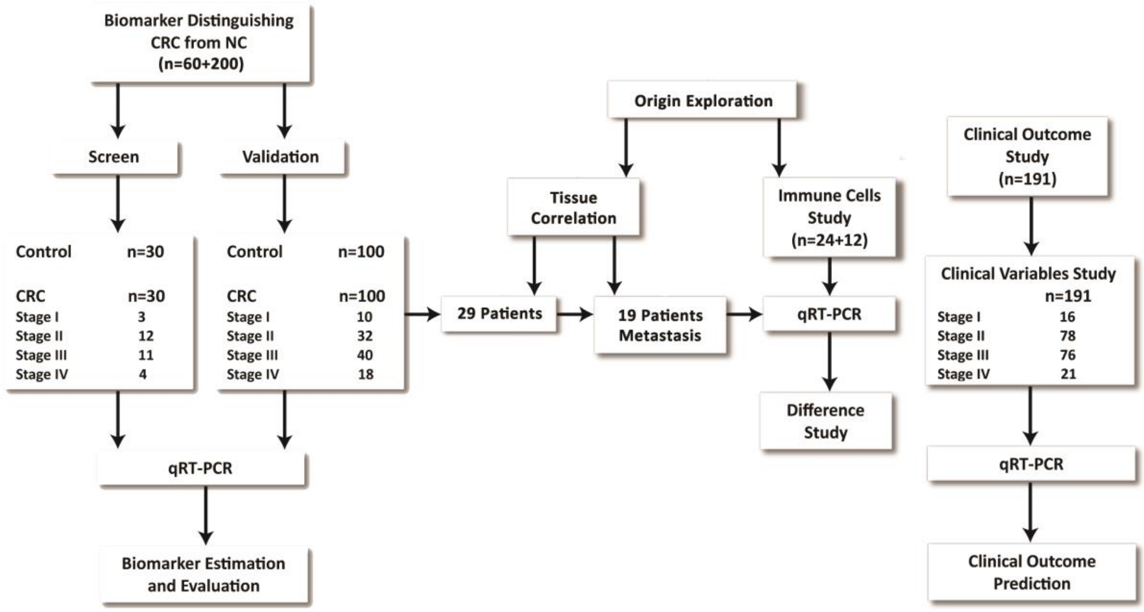

Figure S3. Study design. The blood gene expression was identified using a screening set and an independent validation set. Blood from another 191 patients was detected for clinical outcomes. Primary lesion and metastasis were also investigated. Another group of blood were detected on immune cells.
