# Supplementary material for: Nuclear-enriched abundant transcript 1 as a diagnostic and prognostic biomarker in colorectal cancer
Source: Mol Cancer. 2015 Nov 9;14:191. doi: 10.1186/s12943-015-0455-5 (PMC4640217; doi:10.1186/s12943-015-0455-5)
Supplement: Additional file 6: Table S3. — Characteristics of the CRC and control populations. (PDF 183 kb) [file 12943_2015_455_MOESM6_ESM.pdf]

Table S3. Characteristics of the CRC and control populations.

Supplementary Table S1

Characteristics of the CRC and control populations							
Variable	Screening		Validation		Clinical Variables Study	Immune Cells Study	
	CRC n=30	NC n=30	CRC n=100	NC n=100	CRC n=191	CRC n=24	NC n=12
Age,y							
Mean	56.1	56.3	56.3	56.2	55.5	51.1	47.6
Range	27-78	38-74	36-78	32-74	24-77	32-72	25-56
Gender, no. (%)							
Male	15(50.0)	15(50.0)	54(54.0)	35(35.0)	101(52.8)	12(50.0)	6(50.0)
Female	15(50.0)	15(50.0)	46(46.0)	65(65.0)	90(47.2)	12(50.0)	6(50.0)
Tumor Site, no. (%)							
Colon	10(33.3)		43(43.0)		59(30.9)	10(41.7)	
Rectum	20(66.7)		57(57.0)		132(69.1)	14(58.3)	
Tumor Stage, no. (%)							
Stage I	3(10.0)		10(10.0)		16(8.3)	6(25.0)	
Stage II	12(40.0)		32(32.0)		78(40.8)	6(25.0)	
Stage III	11(36.7)		40(40.0)		76(39.8)	6(25.0)	
Stage IV	4(13.3)		18(18.0)		21(11.1)	6(25.0)	
