# Supplementary material for: Nuclear-enriched abundant transcript 1 as a diagnostic and prognostic biomarker in colorectal cancer
Source: Mol Cancer. 2015 Nov 9;14:191. doi: 10.1186/s12943-015-0455-5 (PMC4640217; doi:10.1186/s12943-015-0455-5)
Supplement: Additional file 7: Table S4. — Primer sequences for NEAT1 and reference genes. (PDF 132 kb) [file 12943_2015_455_MOESM7_ESM.pdf]

Table S4. Primer sequences for NEAT1 and reference genes

NEAT1_v1	
Forward	GTAGTTCAGTTCTTAACCAATG
Reverse	CCATACAGAGCAACATACC
NEAT1_v2	
Forward	CAGAGACACAGGCATTCA
Reverse	GACTACACTCCTTGGTAACT
DECR1	
Forward	CGATGCTACCACCTAATAGT
Reverse	TAGGCTGGACAGAAGAGT
β-ACTIN	
Forward	TGTCCACCTTCCAGCAGAT
Reverse	GCCATGCCAATCTCATCTTGT
